# Toward Healthier Cookie Habits: Assessing the Role of Packaging Visual Appearance in the Expectations for Dietary Cookies in Digital Environments

**DOI:** 10.3389/fpsyg.2021.679443

**Published:** 2021-07-22

**Authors:** Felipe Reinoso-Carvalho, Raffaele Campo, Modesto De Luca, Carlos Velasco

**Affiliations:** ^1^Universidad de Los Andes School of Management, Bogotá, Colombia; ^2^Department of Economics, Management and Business Law, University of Bari, Bari, Italy; ^3^Independent Researcher, Rome, Italy; ^4^Centre for Multisensory Marketing, Department of Marketing, BI Norwegian Business School, Oslo, Norway

**Keywords:** crossmodal correspondences, dietary cookies, expectation, packaging, well-being

## Abstract

As we tend to consume more and more via e-commerce platforms, the digital version of a dietary product’s package can be one of the most important touchpoints that the consumer has with such product during the purchasing stage of the consumer’s journey. Hence, a dietary food/drink properly presented via its packaging in e-commerce is key, for example, to nudge consumers toward healthier purchase habits. In this study, we assessed the role of different configurations of visual cues commonly present in a product’s packaging (jar vs. bag, transparent vs. opaque, labeled vs. unlabeled) in the expectations associated with dietary cookies when presented in a digital environment. A between-participants study was conducted where eight different packages with different combinations of the three aforementioned features were digitally evaluated by the participants. The results suggest that the presence (vs. absence) of labeling triggered the highest ratings on most assessed dimensions (product quality, healthiness, lightness, sweetness, crumbliness, price, tastiness, greediness for product, product/packaging liking). Moreover, transparent (vs. opaque) packaging tends to yield higher expectations concerning this product’s quality (i.e., product liking, package liking, greediness), though it has an opposite effect on the expected healthiness for such cookies. Some particular interactions between these three visual cues were also observed and are discussed as part of the obtained results. In summary, our results point to how the visual appearance of packaging can be strategically used in order to potentially nudge consumers toward healthier cookie purchase habits.

## Introduction

Packaging may be regarded as the “visiting card” of a food/drink product, since it is the first element that usually catch a consumer’s attention in, for example, a retail environment, acting as a key interface between brands and consumers. From the perspective of food marketing, the packaging of a food/drink is, in fact, an essential tool for communicating and promoting a product (Hawkes, 2010; Küster et al., 2019). Decades ago, Guss (1967) already suggested that “packaging is marketing.” That said, it is evident that packaging is not only about containing, protecting, and conserving foods and drinks, but it is a crucial element to be taken into consideration during the experience of such type of products (Krishna et al., 2017). Indeed, it has been suggested that packaging is a multisensory device capable of delivering specific consumer experiences (Velasco and Spence, 2019). The visual appearance of packaging, for instance, seems to be one of the most important elements during the formation of the expectations concerning the experience of the food/drink being contained in such packaging (Pinson, 1986; Selame and Koukos, 2002; Fenko et al., 2010; Spence and Velasco, 2018).

Indeed, it has been extensively shown that consumers’ decisions are significantly affected by the visual appearance of a product, including its packaging (Imram, 1999; Bloch et al., 2003; Crilly et al., 2004; Folkes and Matta, 2004; Zellner et al., 2004; Creusen and Schoormans, 2005; Schifferstein and Cleiren, 2005; Schifferstein, 2006; Schifferstein and Desmet, 2007; Vieira, 2010). Nowadays these reflections seem to grow in importance, as we tend to consume more and more via e-commerce platforms, and where the 2D, 3D, or augmented reality (AR) digital version of a product’s package is, most of the time, the single interaction that the consumer has with such product during the purchasing decision process (Petit et al., 2019, 2021). A food/drink properly presented via its packaging in e-commerce may be key, for example, to nudge consumers toward choosing new products. Packaging may also be critical for communicating health-related information (Vith et al., 2010; Miklavec et al., 2016; Hagmann and Siegrist, 2020), and where dietary food products may be important choices during the corresponding decision-making processes.

With the above being said, the general purpose of this study consisted of evaluating the role of a packaging’s type, label, and transparency on dietary products expectations. In particular, we aimed at looking for ways to customize the online experience of dietary cookies more accurately via its packaging. That said, it would be possible to nudge consumers more effectively toward healthier purchase choices, while at the same time allowing companies to better market dietary food products.

### Theoretical Framework

#### The Choice of Food Stimuli

Dietary cookies were chosen as the product for this experiment. Cookies can be acquired in wide variety of types when it comes to flavor and production process (i.e., from fully crafted, till mass-produced), as well as concerning dietary properties (e.g., from very high-caloric till very specific choices in terms of dietary conditions). At present, cookies are, in fact, one of the most consumed products during breakfast in some European countries, such as Italy^1^. What is more, the particular market size of dietary cookies is expected to expand at 5.0% rate between 2019 and 2025, worldwide, and where the distribution via online channels is also expected to grow at 5.2% rate between 2019 and 2025 (Market Analysis Report, 2019). In summary, assessing the experience of dietary cookies, digitally, would certainly be in line with the demands of the present and future market.

#### Verbal Typologies in Packaging

When it comes to the particularities of the visual appearance of packaging, Silayoi and Speece (2004, 2007) suggested that one may classify visual elements of packaging into two main typologies: verbal (i.e., including health and nutritional claims), and non-verbal (as in color, type, shape, size, graphics).

Concerning verbal typologies, several studies have verified how particular sensory aspects of products, which may also be related to healthiness, can be influenced by messages present on food/beverage packaging (e.g., Tuorilla and Cardello, 2002; Verbeke et al., 2009; Carrillo et al., 2012; Lwin et al., 2014; Miraballes et al., 2014; Pinto et al., 2017; Steinhauser and Hamm, 2018; Steinhauser et al., 2019). Carrillo et al. (2012), for instance, studied consumers’ perceptions of low-calorie cookies packaging, alternating tasting/non-tasting sessions with presence vs. absence of information on packaging. They found that participants paid more attention to short nutritional information, while also reporting negative evaluations associated to excessive information on packaging. Meanwhile, three versions of a snack-bar were presented by Pinto et al. (2017) to Brazilian consumers (the first version with no information at all, the second with the product’s packaging, and the third with the product’s packaging including health-related information). The latter findings suggest that packaging attributes, along with price/flavor/health information, can influence consumers in their choice to purchase a snack bar, while also having a positive impact on consumers’ product acceptance. Efficacy on health claims have also been analyzed by Lwin et al. (2014). Their study shed light on the apparent low-impact of nutritional claims on unrestrained eaters, which contrasted with restrained eaters, whose opinion on not-so-healthy food improved when the corresponding package contained information relative to healthiness.

With the above being said, in this study, we decided to assess the effect of verbal typologies in packaging on the expectations of dietary cookies in a digital environment, by manipulating presence vs. absence of labeling.

#### Non-verbal Typologies in Packaging

When it comes to non-verbal typologies, existing evidence points toward crossmodal correspondences (that is, the associations that people make between features across the senses; Spence, 2011) as playing a crucial role on how the visual appearance of packaging can significantly affect our expectations, and consequent tasting experience of foods and drinks (Reinoso Carvalho et al., 2017a; Velasco and Spence, 2019). Based on crossmodal correspondences, different studies have revealed the role of a food/drink packaging and/or general container (i.e., cups), not only in terms of expectations, but also in actual taste/flavor perception (e.g., Raudenbush et al., 2002; Letona et al., 2014; van Rompay et al., 2014; Tu et al., 2015; Tijssen et al., 2017; Mead and Richerson, 2018; Spence and Carvalho, 2019).

For instance, shape of packaging has been shown to affect the perception of different food/drink (e.g., Ares and Deliza, 2010; Becker et al., 2011; Velasco et al., 2014, 2016; Spence and Wan, 2015; Simmonds et al., 2019). The impact of the shape of a yogurt’s packaging, for example, was analyzed by Becker et al. (2011), where participants sensitive to design perceived the yoghurt’s flavor as more intense when packed in an angular packaging, as compared to a rounder one. Concerning dietary food, packaging shape also seems to moderate perceived healthiness (e.g., Fenko et al., 2016; Festila and Chrysochou, 2018; Yarar et al., 2019; Ikonen et al., 2020). In particular, Yarar et al. (2019) showed that a slim humanoid-shaped type of package persuaded women with normal-to-high body-mass-index that such package contained healthier food, when compared to less slim packaging (cf. van Ooijen et al., 2017; Fenko, 2019). Koo and Suk (2016) also verified that food in taller packages tend to be perceived as having fewer calories than when contained in wider and less-tall packages. Marques da Rosa et al. (2018) also observed that healthiness of buttery cookies is deemed higher in case of a rounded, and red-to-yellow packages, compared to angular and blue-to-green ones.

Moreover, transparency (vs. opaqueness) of packaging is a critical attribute that has also been shown to influence food and drink expectations and experiences (Deng and Srinivasan, 2013; Adam and Ali, 2014; Tu et al., 2015; Simmonds and Spence, 2017; Simmonds et al., 2018; Sabri et al., 2020). For example, Tu et al. (2015) observed that cold tea tends to be perceived as sweeter when served in a transparent glass cup, as compared to when poured in an opaque paper/plastic receptacle. Adam and Ali (2014) also conducted a comparison of type of milk packaging. Here, they found a positive correlation between purchase intention and a transparent glass-milk container, and a negative correlation of the purchase intention associated to the opaque tetra-pak, and plastic milk containers. In this study, the presence of nutritional information on the package’s labeling also increased purchase intention.

In the present research, we focused on the role of packaging type and transparency on the expectations associated with dietary food products in a digital environment. Type and transparency are ubiquitous in packaging, while being both widely used to differentiate brands in the marketplace. In particular, here, we thought of the fact that, nowadays, most cookies are actually commercialized in angular-shaped, and opaque bag types (e.g., Robertson, 2011; Davidson, 2018). Nevertheless, we also recalled the most utilitarian cylindrical/round shaped transparent cookie jar types, which were often found as part of households in the United States, Canada, and United Kingdom (in the latter they are perhaps most commonly known as biscuit jars; Franklin, 1979; Allen, 2004). In brief, when it comes to non-verbal typologies in packaging, we were interested in comparing how such specific types of packaging (bag vs. jar, with both containing specific shapes), at contrasting levels of transparency, could impact the expectations of consumers for dietary cookies.

### Research Gap

A recent study conducted by De Luca et al. (2019) served as principal inspiration for the present research. In this study it was assessed how consumers perceived aspects of dietary cookies when served in an unlabeled transparent-round-glass jar (as in a cookie jar), as compared to when served it its commercial/original packaging (in this case, an opaque bag which included labeling with branding and formulation; cf. Adam and Ali, 2014). These results, based on 31 European participants, suggested that such differences in packaging appearance may prompt different expectations on dietary cookies. For instance, this study reported that dietary cookies were thought as potentially lighter, but not necessarily healthier, when being offered in their original labeled packaging, as compared to when offered in the unlabeled transparent jar. Moreover, the participants rated the same cookies as potentially more caloric when presented in the unlabeled jar, as compared to the ratings under the influence of the original packaging. The obtained evidence also suggested that ‘lightness’ and ‘calories’ related wording were more effective message carriers for dietary cookies labeling, rather than ‘healthy’ type of wording, or wording related to flavor/hedonic sensations elicited by the cookies experience (i.e., crumbliness, greediness).

Based on De Luca et al.’s (2019) assessment, we decided to design this new study focused on carefully evaluating the effects of packaging transparency and type on expectations in an experiment resembling e-commerce. The latter is also aligned with the e-commerce growing trend for this food category (e.g., Market Analysis Report, 2019). As such, our new study would be principally about the expectations elicited by the digital visual appearance of packaging, where tasting the cookies and touching the packaging would not be part of our experimental scope (cf. Citrin et al., 2003; Overmars and Poels, 2015; Petit et al., 2019).

Here, we also saw the need to use customized brand-free labeling (something that was not considered in the aforesaid study). In fact, while designing our own experimental label for this new study, we would be able to optimize the amount and type of information present in such labeling (cf. Carrillo et al., 2012). The latter is relevant when assuming that the usual e-commerce platform does not always allow the consumer to properly read all the information that may be present in a dietary product’s label. Finally, we wanted to more accurately control the three dimensions of a packaging appearance that were explored in the latter study as well (jar vs. bag, transparency vs. opaqueness, unlabeled vs. labeled packaging). For this, we decided to compare such dimensions not only between two experimental conditions (as in De Luca et al., 2019), but across eight different versions of packaging.

### The Present Study

The objective of this study, therefore, was to look for ways to customize the online experience of dietary cookies more accurately via its packaging. For this, we assessed how different combinations of three key aspects of a packaging appearance (with vs. without labeling, bag vs. jar, transparency vs. opaqueness) would affect the expectations of consumers while evaluating dietary cookies in a 2D digital context.

First, we hypothesized that an optimized label mainly communicating the dietary attributes on the packaging would be more strongly associated with healthier type of cookies, when compared to an unlabeled version of the same packaging. Second, we thought of comparing two packaging types (a cookie jar and a bag). Specifically, we thought that a jar usually has rounder shapes than most cookie bags (Robertson, 2011; Davidson, 2018). Based, for instance, on crossmodal correspondences research – which suggests that round shapes are more naturally associated with sweet/smooth sensations, when compared to more angular shapes (cf. Becker et al., 2011; see also Reinoso Carvalho et al., 2017b, for an example), one may expect that a jar may prompt higher expectations toward a sweeter, and/or smoother, type of cookie, when compared to a bag. Third, a transparent packaging would allow the cookies to be fully exposed, and where we hypothesized that this would have positive consequences on the expectations of consumers while evaluating the product (Petit et al., 2021; Simmonds and Spence, 2017; Simmonds et al., 2018). The latter may be even more relevant in a digital environment, where none of the senses, besides vision, tend to be significantly activated (at least not yet).

In brief, by contrasting the appearance of a packaging across such three dimensions, we tested the following main general hypothesis:

*H1: Changes in the visual appearance of packaging, in terms of presence (vs. absence) of label, packaging type (jar vs. bag), and transparency (vs. opaqueness) will modulate the expectations for dietary cookies in digital environments.*

The test of the general main hypothesis was carried out across the following three specific hypothesis, and where each of these particular hypotheses focused on the three specific aspects of the packaging being manipulated:

*H1a: The presence vs. absence of labeling will principally modulate aspects related to the expected healthiness for this product (calories, healthiness, lightness).*

*H1b: A jar will most likely trigger sweeter and/or smoother sensations for the cookies, when compared to a bag type of packaging.*

*H1c: A transparent packaging will prompt higher expectations concerning specific sensory (e.g., sweetness, crumbliness) and qualitative (e.g., greediness, preference, price) aspects of cookies flavor.*

In general, we assumed that the said modulation in expectations would be measurable in terms of the ratings related to healthiness (e.g., lighter, healthier, caloric content), differences in hedonic/qualitative ratings (e.g., tastiness, greediness – as in ‘these cookies look so good they make me hungry,’ price), and/or differences in sensory flavor ratings (e.g., sweetness, crumbliness).

## Materials and Methods

### Participants

In total, 496^2^ participants took part in the experiment (*n* = 496; 62.50% males, Mean of age 25.71 years *SD* = 7.91). The survey was programmed on Qualtrics software^3^ and the participants were recruited on Prolific Academic^4^, in exchange for GBP 0.63. The focus on this recruitment was to model the general population, so no specific filters were set concerning the attitudes that the sampled population might have toward cookies or dietary cookies. Here, most participants reported eating cookies (95.00%), loving cookies (86.10%), and usually paying attention to food/drink product’s labeling while shopping (73.80%). Only 31.50% of the participants reported actually consuming light and/or dietary type of cookies. Concerning cookies eating habits, the obtained sample appears as representative of an European population. For instance, previous evidence has reported that 96% of such consumers tend to buy into this category, and that cookies with certain dietary characteristics are being consumed by more or less a third of this kind of population (Harris Interactive Survey, 2019).

### Stimuli

**FIGURE 1:**
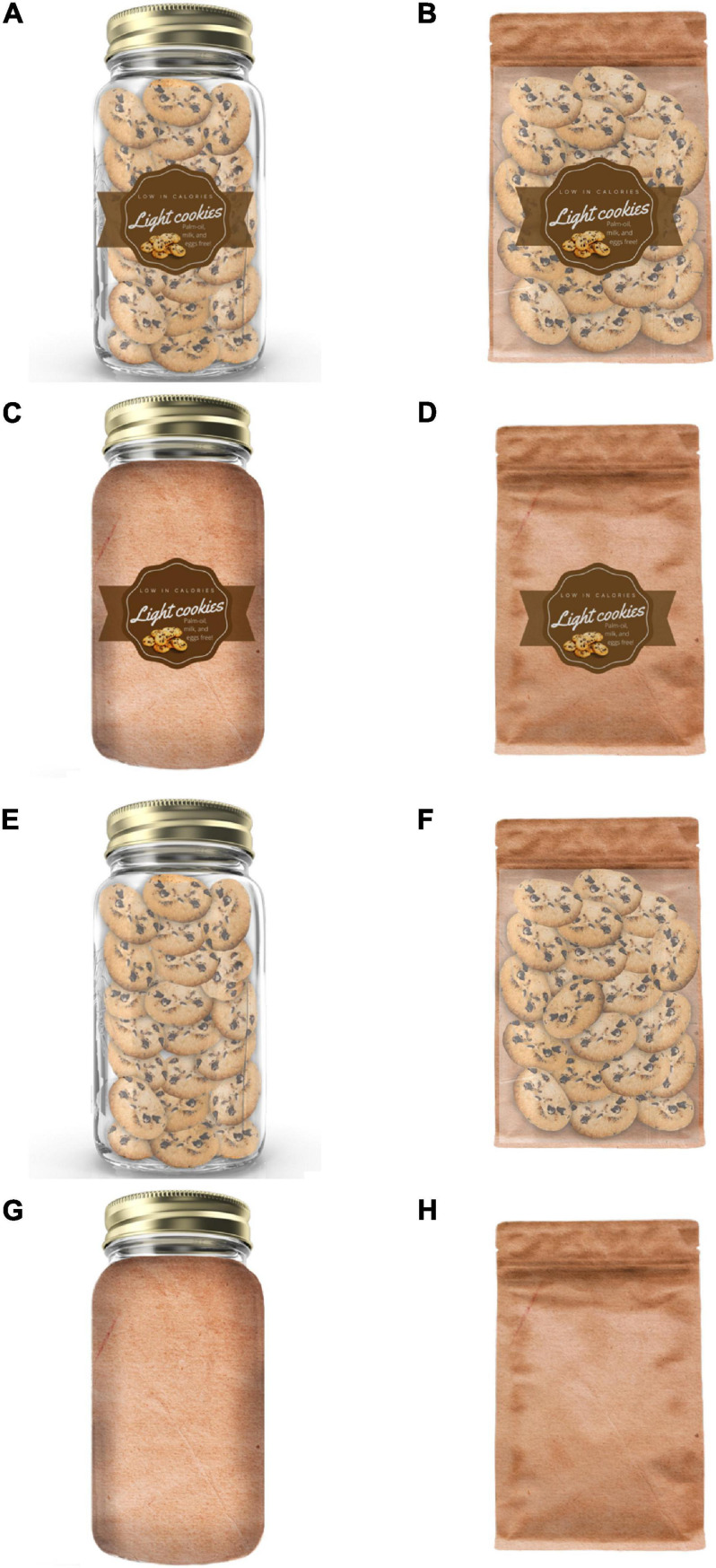
The eight experimental packages [labeled transparent jar **(A)**, labeled transparent bag **(B)**, labeled opaque jar **(C)**, labeled opaque bag **(D)**, unlabeled transparent jar **(E)**, unlabeled transparent bag **(F)**, unlabeled opaque jar **(G)**, unlabeled opaque bag **(H)**]. The packages were presented scaled to match in size, and large enough in order for the participants be able to clearly see all the available information present in the label [the latter only when applicable, since **(E–H)** versions were unlabeled].

Eight different versions of packaging were produced for this experiment. They were carefully designed with respect to any unintended influences, for example, when it comes to the quality prompted by the packaging type, as well as while keeping away from resembling existing cookies brands. Such eight different versions of packaging included two variations of packaging type (jar vs. bag), two variations of packaging transparency (transparent vs. opaque), and two variations of labeling (labeled vs. unlabeled). Based on Carrillo et al. (2012) and De Luca et al. (2019), the label included the product’s title (in this case, ‘Light cookies’), along with highlights on the product’s formula (‘low in calories’; ‘palm-oil, milk, and eggs free’). Figure 1 shows the different versions of the packaging stimuli.

### Design and Procedure

This study followed a 2 (packaging type: jar vs. bag) × 2 (labeling: label vs. no label) × 2 (transparency: opaque vs. transparent) between-participants experimental design. Each participant was randomly assigned to, and asked to evaluate, only one of the eight versions of the dietary cookies packaging (see Table 1). In particular, they were asked to evaluate the packages in terms of healthiness, quality, greediness, price, lightness, calories, crumbliness, sweetness, tastiness, cookies/packaging liking. The completion of the survey lasted for approximately 5 min in total.

**TABLE 1:** n, mean of age (with corresponding SD), and % of females, for each between-participants packaging sample.

Dietary cookies packaging			*n*	Mean age – years (SD)	% Females
Labeled	Transparency	Format			
Labeled	Transparent	Jar	62	27.31 (9.22)	33.90%
		Bag	63	26.35 (8.38)	33.30%
	Opaque	Jar	62	24.85 (5.77)	33.90%
		Bag	61	24.25 (6.70)	47.50%
Unlabeled	Transparent	Jar	61	26.80 (8.79)	41.10%
		Bag	62	25.88 (8.93)	37.10%
	Opaque	Jar	63	25.04 (6.57)	46.00%
		Bag	62	25.19 (8.16)	29.00%

The packages were presented scaled to match in size (1041 × 1476 pixels), and large enough in order for the participants be able to read all the available information present in the label (the latter only when applicable, since four of the eight versions of packaging were unlabeled; see Figure 1). Since the transparent packaging allowed the participants to actually see the cookies, and the opaque did not, it was decided to include mini replicas of the cookies as part of the label.

The answers concerning the expectations elicited by the packaging were all based on 5-point rating scales (anchored with 1 = ‘Not at all’, 5 = ‘Very much’). After answering these questions, the participants were also asked to provide some demographic information (age, gender), and their general cookies-consumption habits. Appendix Table A, shows a summary of the variables assessed in the survey. The order of the presentations of the survey’s questions, and multiple-choice answers, were fully randomized.

### Data Analysis

A 2 × 2 × 2 multivariate analysis of variance (MANOVA) was conducted via SPSS 26, with type of packaging, labeling, and transparency as between-factors, and the rating-scales as the dependent variables (see Appendix Table A, for an overview on these dependent variables). Gender and age were included as controls (covariates).

## Results

**FIGURE 2:**
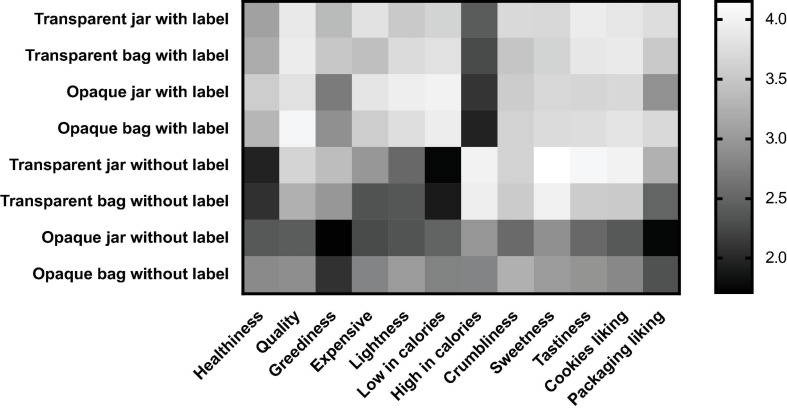
Grayscale heat map representing the mean values of each dependent variables (*X*-axis) across the different experimental conditions (*Y*-axis). The lower mean values are being represented with the darkest colors, and vice-versa.

Table 2 (Means, SD) and Figure 2 (Means) show a descriptive summary of results across the eight different types of packaging.

**TABLE 2:** Summary of the dependent variable means and corresponding standard deviations (SD) as a function of the eight different packaging presentations.

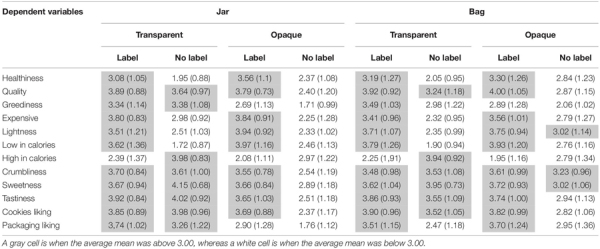

Table 3 shows the details on the general results of the multivariate test. In general, the multivariate tests show a main effect of packaging labeling and transparency (*p* ≤ 0.001 for both), as well as an interaction effect of packaging labeling^∗^transparency (*p* ≤ 0.001), packaging type^∗^transparency (*p* ≤ 0.001), and with all of the three packaging factors combined (*p* = 0.035). No interaction effects with age (*p* = 0.250) or gender (*p* = 0.210) were found.

**TABLE 3:** Summary of the results of the multivariate test, considering all combinations of packaging factors (type – bag vs. jar, transparency – transparent vs. opaque, labeling – with vs. without label), and including controls for gender and age (only Pillai’s trace test being reported).

Multivariate tests^a^									
Effect		Value	*F*	Hypothesis df	Error df	Sig.	$\eta_{\text{p}}^{2}$	Non-cent. Parameter	Observed power^c^
**Intercept**	**Pillai’s Trace**	**0.78**	**141580^b^**	**12.00**	**474.00**	**<0.001**	**0.78**	**1698.96**	**1.00**
Age	Pillai’s Trace	0.03	1250^b^	12.00	474.00	0.250	0.03	15.00	0.71
Gender	Pillai’s Trace	0.03	1305^b^	12.00	474.00	0.210	0.03	15.66	0.74
**Labeling**	**Pillai’s Trace**	**0.46**	**33678^b^**	**12.00**	**474.00**	**<0.001**	**0.46**	**404.14**	**1.00**
Type	Pillai’s Trace	0.03	1180^b^	12.00	474.00	0.290	0.03	14.16	0.68
**Transparency**	**Pillai’s Trace**	**0.25**	**13130^b^**	**12.00**	**474.00**	**<0.001**	**0.25**	**157.57**	**1.00**
Labeling* Type	Pillai’s Trace	0.04	1676^b^	12.00	474.00	0.690	0.04	20.11	0.86
Labeling * Transparency	**Pillai’s Trace**	**0.13**	**5672^b^**	**12.00**	**474.00**	**<0.001**	**0.13**	**68.06**	**1.00**
Type * Transparency	**Pillai’s Trace**	**0.09**	**3720^b^**	**12.00**	**474.00**	**<0.001**	**0.09**	**44.64**	**1.00**
Labeling * Type * Transparency	**Pillai’s Trace**	**0.05**	**1874^b^**	**12.00**	**474.00**	**0.040**	**0.05**	**22.49**	**0.90**

Values in bold indicate a significant different at 95% confidence (*p* ≤ 0.050). ^*a*^Design: intercept + age + gender + labeling + type + transparency + labeling * type + labeling * transparency + type * transparency + labeling * type * transparency. ^*b*^Exact statistic. ^*c*^Computed using α = 0.05.

Table 4 shows the details of the results concerning the between-participants effects. Significant effects are being reported at 95% confidence (*p* ≤ 0.050). Such effects were found for all of the dependent variables concerning packaging labeling (healthiness, quality, greediness, expensive, lightness, high/low in calories, crumbliness, sweetness, tastiness, cookies and packaging liking); all of the dependent variables assessed via packaging transparency (except for expensive); all of the dependent variables when it comes to labeling^∗^transparency interactions (except for healthiness, expensive, and lightness); all of the dependent variables assessed via packaging type^∗^transparency interactions (except for healthiness, greediness, lightness, low/high in calories, and sweetness); and for healthiness, expensive, lightness, tastiness and cookies liking ratings, when it comes to packaging labeling^∗^type^∗^transparency interactions.

**TABLE 4:** Summary of the main effects and interactions.

Tests of between-subjects effects								
**Source**		**Type III sum of squares**	**df**	**Mean square**	***F***	**Sig.**	**$\eta_{\text{p}}^{2}$**	**Observed power^*a*^**

**Intercept**	**Healthiness**	**193.87**	**1.00**	**193.87**	**156.68**	**<0.001**	**0.24**	**1.00**
	**Quality**	**380.76**	**1.00**	**380.76**	**363.44**	**<0.001**	**0.43**	**1.00**
	**Greediness**	**183.95**	**1.00**	**183.95**	**149.11**	**<0.001**	**0.24**	**1.00**
	**Expensive**	**335.80**	**1.00**	**335.80**	**315.61**	**<0.001**	**0.39**	**1.00**
	**Lightness**	**303.47**	**1.00**	**303.47**	**278.24**	**<0.001**	**0.36**	**1.00**
	**Low in calories**	**296.34**	**1.00**	**296.34**	**226.31**	**<0.001**	**0.32**	**1.00**
	**High in calories**	**211.24**	**1.00**	**211.24**	**166.84**	**<0.001**	**0.26**	**1.00**
	**Crumbliness**	**405.05**	**1.00**	**405.05**	**415.05**	**<0.001**	**0.46**	**1.00**
	**Sweetness**	**393.10**	**1.00**	**393.10**	**441.64**	**<0.001**	**0.48**	**1.00**
	**Tastiness**	**378.45**	**1.00**	**378.45**	**361.24**	**<0.001**	**0.43**	**1.00**
	**Cookies liking**	**404.42**	**1.00**	**404.42**	**402.85**	**<0.001**	**0.45**	**1.00**
	**Packaging liking**	**308.73**	**1.00**	**308.73**	**220.98**	**<0.001**	**0.31**	**1.00**
**Labeling**	**Healthiness**	**119.46**	**1.00**	**119.46**	**96.54**	**<0.001**	**0.17**	**1.00**
	**Quality**	**91.95**	**1.00**	**91.95**	**87.77**	**<0.001**	**0.15**	**1.00**
	**Greediness**	**40.02**	**1.00**	**40.02**	**32.44**	**<0.001**	**0.06**	**1.00**
	**Expensive**	**140.33**	**1.00**	**140.33**	**131.90**	**<0.001**	**0.21**	**1.00**
	**Lightness**	**170.67**	**1.00**	**170.67**	**156.48**	**<0.001**	**0.24**	**1.00**
	**Low in calories**	**321.17**	**1.00**	**321.17**	**245.27**	**<0.001**	**0.34**	**1.00**
	**High in calories**	**192.94**	**1.00**	**192.94**	**152.39**	**<0.001**	**0.24**	**1.00**
	**Crumbliness**	**15.83**	**1.00**	**15.83**	**16.22**	**<0.001**	**0.03**	**0.98**
	**Sweetness**	**3.45**	**1.00**	**3.45**	**3.88**	**0.050**	**0.01**	**0.50**
	**Tastiness**	**35.74**	**1.00**	**35.74**	**34.11**	**<0.001**	**0.07**	**1.00**
	**Cookies liking**	**51.55**	**1.00**	**51.55**	**51.35**	**<0.001**	**0.10**	**1.00**
	**Packaging liking**	**127.01**	**1.00**	**127.01**	**90.91**	**<0.001**	**0.16**	**1.00**
**Transparency**	**Healthiness**	**25.38**	**1.00**	**25.38**	**20.51**	**<0.001**	**0.04**	**0.99**
	**Quality**	**19.73**	**1.00**	**19.73**	**18.84**	**<0.001**	**0.04**	**0.99**
	**Greediness**	**112.26**	**1.00**	**112.26**	**90.99**	**<0.001**	**0.16**	**1.00**
	Expensive	0.10	1.00	0.10	0.10	0.760	0.00	0.06
	**Lightness**	**6.53**	**1.00**	**6.53**	**5.98**	**0.010**	**0.01**	**0.68**
	**Low in calories**	**32.74**	**1.00**	**32.74**	**25.00**	**<0.001**	**0.05**	**1.00**
	**High in calories**	**55.67**	**1.00**	**55.67**	**43.97**	**<0.001**	**0.08**	**1.00**
	**Crumbliness**	**15.37**	**1.00**	**15.37**	**15.75**	**<0.001**	**0.03**	**0.98**
	**Sweetness**	**34.42**	**1.00**	**34.42**	**38.67**	**<0.001**	**0.07**	**1.00**
	**Tastiness**	**48.15**	**1.00**	**48.15**	**45.96**	**<0.001**	**0.09**	**1.00**
	**Cookies liking**	**50.75**	**1.00**	**50.75**	**50.56**	**<0.001**	**0.09**	**1.00**
	**Packaging liking**	**42.23**	**1.00**	**42.23**	**30.23**	**<0.001**	**0.06**	**1.00**
Labeling * Transparency	Healthiness	2.97	1.00	2.97	2.40	0.120	**0.00**	**0.34**
	**Quality**	**20.03**	**1.00**	**20.03**	**19.12**	**<0.001**	**0.04**	**0.99**
	**Greediness**	**12.90**	**1.00**	**12.90**	**10.46**	**<0.001**	**0.02**	**0.90**
	Expensive	1.46	1.00	1.46	1.38	0.240	**0.00**	**0.22**
	Lightness	0.01	1.00	0.01	0.01	0.920	0.00	0.05
	**Low in calories**	**8.57**	**1.00**	**8.57**	**6.54**	**0.010**	**0.01**	**0.72**
	**High in calories**	**19.05**	**1.00**	**19.05**	**15.04**	**<0.001**	**0.03**	**0.97**
	**Crumbliness**	**14.08**	**1.00**	**14.08**	**14.42**	**<0.001**	**0.03**	**0.97**
	**Sweetness**	**39.71**	**1.00**	**39.71**	**44.61**	**<0.001**	**0.08**	**1.00**
	**Tastiness**	**22.97**	**1.00**	**22.97**	**21.93**	**<0.001**	**0.04**	**1.00**
	**Cookies liking**	**32.79**	**1.00**	**32.79**	**32.66**	**<0.001**	**0.06**	**1.00**
	**Packaging liking**	**7.83**	**1.00**	**7.83**	**5.60**	**0.020**	**0.01**	**0.66**
Type * Transparency	Healthiness	0.00	1.00	0.00	0.00	0.980	0.00	0.05
	**Quality**	**8.42**	**1.00**	**8.42**	**8.04**	**<0.001**	**0.02**	**0.81**
	**Greediness**	**4.73**	**1.00**	**4.73**	**3.84**	**0.050**	**0.01**	**0.50**
	**Expensive**	**13.33**	**1.00**	**13.33**	**12.53**	**<0.001**	**0.03**	**0.94**
	Lightness	1.58	1.00	1.58	1.45	0.230	**0.00**	**0.22**
	Low in calories	0.03	1.00	0.03	0.02	0.890	0.00	0.05
	High in calories	0.14	1.00	0.14	0.11	0.740	0.00	0.06
	**Crumbliness**	**8.57**	**1.00**	**8.57**	**8.78**	**<0.001**	**0.02**	**0.84**
	Sweetness	1.49	1.00	1.49	1.67	0.200	**0.00**	**0.25**
	**Tastiness**	**8.50**	**1.00**	**8.50**	**8.11**	**<0.001**	**0.02**	**0.81**
	**Cookies liking**	**7.79**	**1.00**	**7.79**	**7.76**	**0.010**	**0.02**	**0.79**
	**Packaging liking**	**43.81**	**1.00**	**43.81**	**31.36**	**<0.001**	**0.06**	**1.00**
Labeling * Type * Transparency	**Healthiness**	**4.58**	**1.00**	**4.58**	**3.70**	**0.050**	**0.01**	**0.48**
	Quality	3.56	1.00	3.56	3.39	0.070	**0.01**	**0.45**
	Greediness	4.33	1.00	4.33	3.51	0.060	**0.01**	**0.46**
	**Expensive**	**9.09**	**1.00**	**9.09**	**8.54**	**<0.001**	**0.02**	**0.83**
	**Lightness**	**11.89**	**1.00**	**11.89**	**10.90**	**<0.001**	**0.02**	**0.91**
	Low in calories	0.82	1.00	0.82	0.63	0.430	**0.00**	**0.12**
	High in calories	0.20	1.00	0.20	0.16	0.690	0.00	0.07
	Crumbliness	1.62	1.00	1.62	1.66	0.200	**0.00**	**0.25**
	Sweetness	0.40	1.00	0.40	0.45	0.500	0.00	0.10
	**Tastiness**	**4.28**	**1.00**	**4.28**	**4.09**	**0.040**	**0.01**	**0.52**
	**Cookies liking**	**5.58**	**1.00**	**5.58**	**5.55**	**0.020**	**0.01**	**0.65**
	Packaging liking	0.77	1.00	0.77	0.55	0.460	**0.00**	**0.11**
Error	Healthiness	600.13	485.00	1.24				
	Quality	508.10	485.00	1.05				
	Greediness	598.34	485.00	1.23				
	Expensive	516.02	485.00	1.06				
	Lightness	528.98	485.00	1.09				
	Low in calories	635.07	485.00	1.31				
	High in calories	614.07	485.00	1.27				
	Crumbliness	473.31	485.00	0.98				
	Sweetness	431.70	485.00	0.89				
	Tastiness	508.10	485.00	1.05				
	Cookies liking	486.89	485.00	1.00				
	Packaging liking	677.59	485.00	1.40				

*Values in bold indicate a significant different at 95% confidence (*p* ≤ 0.050). ^*a*^Computed using α = 0.05.*

### Main Effect of Packaging Labeling (With vs. Without Label)

Table 5 reports mean differences when comparing general presence vs. absence of label, and regardless of packaging type (jar vs. bag), and level of transparency (transparent vs. opaque). The variables being here considered are those which showed a significant difference in Table 4. This evidence points toward the presence of label in the packaging as triggering higher ratings in all of the assessed dimensions (except for high in calories, which had an opposite direction).

**TABLE 5:** Mean differences for presence vs. absence of label and regardless of type (jar vs. bag) and level of transparency (transparent vs. opaque) of the packaging.

Dependent variable	Mean difference (Labeled-unlabeled)
Healthiness	0.98
Quality	0.86
Greediness	0.57
Expensive	1.07
Lightness	1.18
Low in calories	1.61
High in calories	–1.25
Crumbliness	0.36
Sweetness	0.17
Tastiness	0.54
Cookies liking	0.65
Packaging liking	1.01

*The variables being reported are those that prompted a significant difference in Table 4.*

### Main Effect of Packaging Transparency (Transparency vs. Opaqueness)

Table 6 shows the mean differences when comparing general transparency vs. opaqueness, and regardless of packaging type (jar vs. bag) and labeling (presence vs. absence). The variables being considered are those that prompted a significant difference in Table 4. Here, the evidence shows that a transparent packaging prompted higher ratings for quality, greediness, high in calories, crumbliness, sweetness, tastiness, cookies, and packaging liking ratings. On the other hand, an opaque packaging prompted higher ratings for healthiness, lightness, and low calories.

**TABLE 6:** Mean differences for general transparency vs. opaqueness, and regardless of type (jar vs. bag) and labeling (presence vs. absence) of the packaging.

Dependent variable	Mean difference (Transparency – opaqueness)
Healthiness	–0.46
Quality	0.40
Greediness	0.96
Lightness	–0.02
Low in calories	–0.05
High in calories	0.68
Crumbliness	0.36
Sweetness	0.53
Tastiness	0.63
Cookies liking	0.65
Packaging liking	0.59

*The variables being reported are those that prompted a significant difference in Table 4.*

### Interaction Between Labeling and Transparency

**FIGURE 3:**
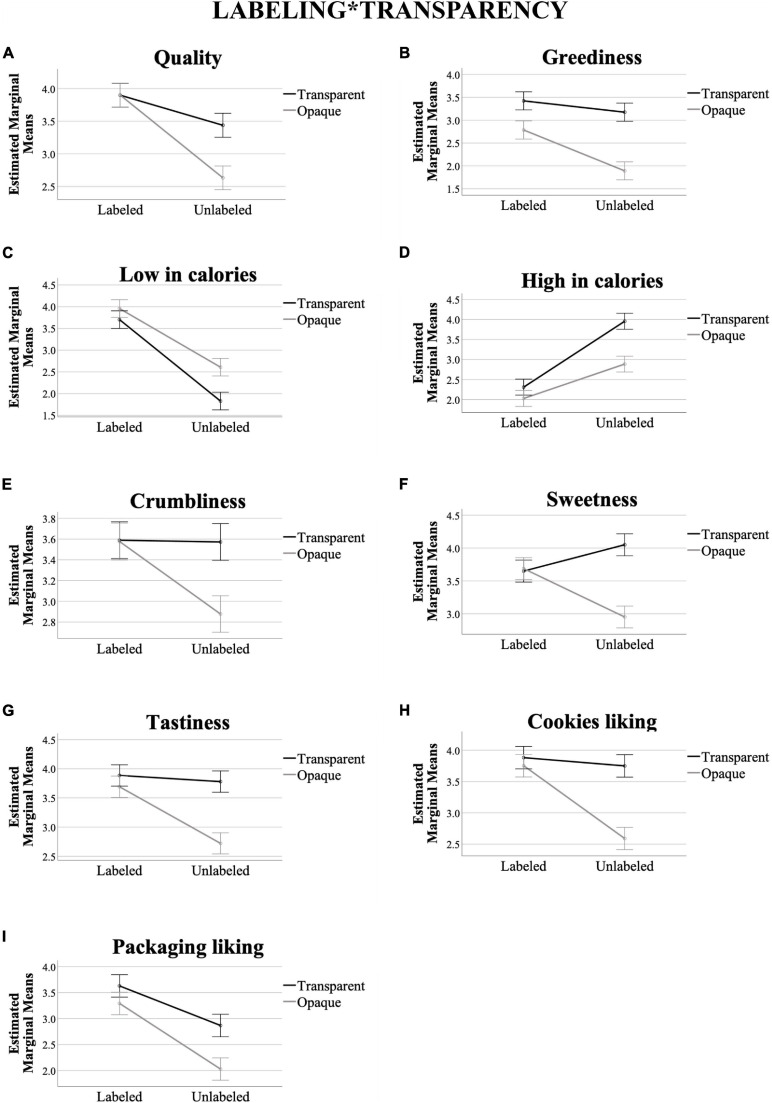
Interaction effects of Labeling*transparency in packaging, across the variables that prompted significant differences at 95% confidence in Table 4 [**(A)** Quality, **(B)** Greediness, **(C)** Low in calories, **(D)** High in calories, **(E)** Crumbliness, **(F)** Sweetness, **(G)** Tastiness, **(H)** Cookies liking, **(I)** Packaging liking]. *Y*-axis are the marginal means. *X*-axis are the labeling conditions. The black (darker) line corresponds to the transparent packaging values, whereas the gray (clearer) line the opaque ones. Error bars show confidence interval at 95%.

As shown in Tables 3, 4, there was evidence of a significant interaction between labeling and transparency on quality, greediness, high/low in calories, crumbliness, sweetness, tastiness, cookies, and packaging liking ratings (Figure 3). In particular, the ratings of quality, greediness, low in calories, and packaging liking, tend to decrease without labeling, and regardless of packaging transparency. Comparing to the latter, high in calories ratings have an opposite direction of effects. For crumbliness, tastiness, and cookies liking ratings, the ratings tend to decrease without labeling, but only for the opaque version of packaging. Concerning sweetness, evidence shows that the label tends to trigger similar effects on transparent and opaque packaging. However, the expected sweetness ratings tend to be enhanced for unlabeled transparent packaging and diminished in the unlabeled opaque one.

### Interaction Between Type and Transparency

**FIGURE 4:**
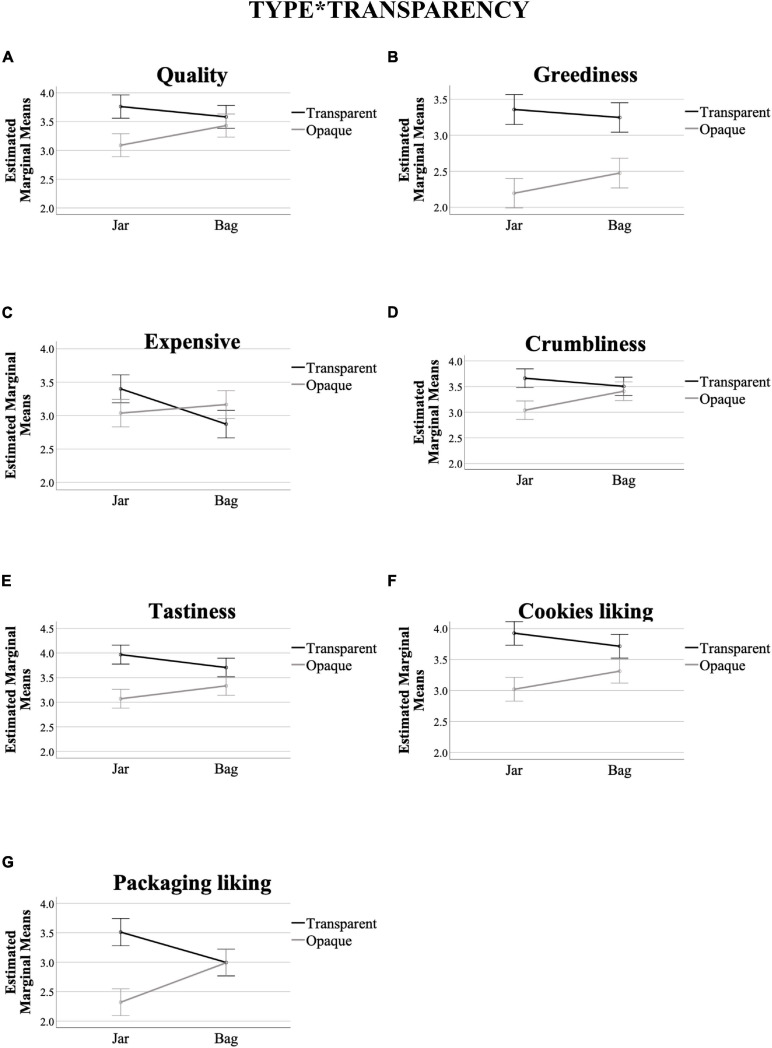
Interaction effects of type*transparency in packaging, across the variables that prompted significant differences at 95% confidence in Table 4 [**(A)** Quality, **(B)** Greediness, **(C)** Expensive, **(D)** Crumbliness, **(E)** Tastiness, **(F)** Cookies liking, **(G)** Packaging liking]. *Y*-axis are the marginal means. *X*-axis are the type conditions. The black (darker) line corresponds to the transparent packaging values, whereas the gray (clearer) line the opaque ones. Error bars show confidence interval at 95%.

Tables 3, 4 show a significant interaction between type and transparency in packaging on quality, greediness, expensive, crumbliness, tastiness, cookies, and packaging liking ratings (see Figure 4). In particular, quality, greediness, crumbliness, tastiness, cookies, and packaging liking ratings tend to increase in the transparent, and decrease in the opaque, jar conditions. For the bag, however, ratings did not seem to be as significantly affected by changes in transparency, when compared to the jar. Concerning expensiveness ratings, people tend to expect the cookies as more expensive when presented in the transparent jar, as compared to the transparent bag. In addition, expensiveness ratings did not seem to be so significantly affected in the opaque version of both packaging.

### Interaction Between Labeling, Type, and Transparency

Tables 3, 4 show evidence of a significant interaction between labeling^∗^type^∗^transparency, concentrated on healthiness, expensive, lightness, tastiness, and cookies liking ratings. Since no significant effects were reported in Table 3 for labeling^∗^type, we decided to independently assess the moderating effect of type of packaging on labeling^∗^transparency, and the moderating effect of labeling on type^∗^transparency of packaging (Gignac, 2019).

#### Moderating Effect of Type of Packaging on Labeling^∗^Transparency

**FIGURE 5:**
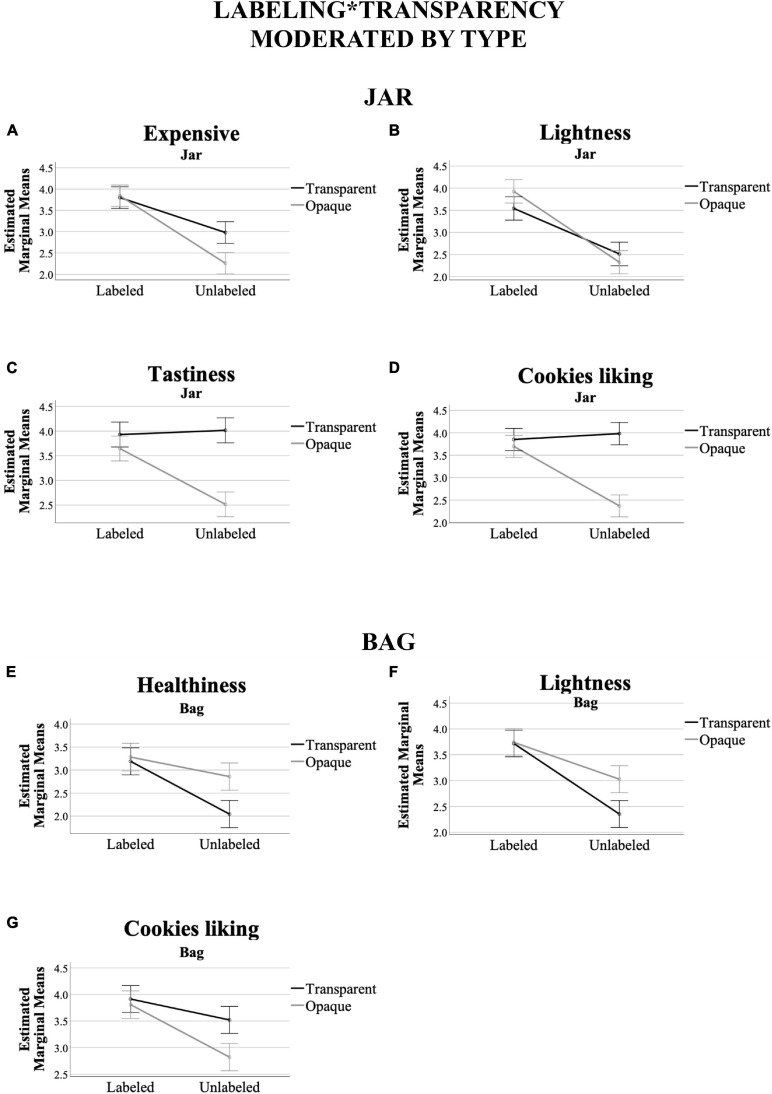
Interaction effect of labeling*transparency, while moderated by type of packaging (jar vs. bag), across the variables that prompted significant differences at 95% confidence in “Moderating Effect of Type of Packaging on Labeling*Transparency” [for jar, **(A)** expensive, **(B)** lightness, **(C)** tastiness, **(D)** cookies liking ratings; for bag, **(E)** healthiness, **(F)** lightness, **(G)** cookies liking ratings]. *Y*-axis are the marginal means. *X*-axis are the labeling conditions. The black (darker) line corresponds to the transparent packaging ratings, whereas the gray (clearer) line the opaque ones. Error bars show confidence interval at 95%.

A significant effect was found for labeling^∗^transparency interaction for both types of packaging (jar - *F*[5,238] = 8.14, *p* < 0.001, η^2^ = 0.146; bag – *F*[5,238] = 3.65, *p* = 0.003, η^2^ = 0.071). This between participants test showed significant effects for the jar on expensive (*F*[1,241] = 8.91, *p* = 0.003, η^2^ = 0.036), lightness (*F*[1,241] = 5.01, *p* = 0.026, η^2^ = 0.020), tastiness (*F*[1,241] = 23.27, *p* < 0.001, η^2^ = 0.088), and cookies liking ratings (*F*[1,241] = 33.83, *p* < 0.001, η^2^ = 0.123) – healthiness (*F*[1,241] = 0.067, *p* = 0.796, η^2^ = 0.000). Concerning the bag, healthiness (*F*[1,242] = 5.73, *p* = 0.017, η^2^ = 0.023), lightness (*F*[1,242] = 5.98, *p* = 0.015, η^2^ = 0.024), and cookies liking ratings (*F*[1,242] = 5.17, *p* = 0.024, η^2^ = 0.021) showed significant effects - expensive (*F*[1, 242] = 1.56, *p* = 0.213, η^2^ = 0.006); tastiness (*F*[1,242] = 2.95, *p* = 0.087, η^2^ = 0.012). The labeling^∗^transparency interaction moderated by type of packaging is visualized in Figure 5. In brief, the moderating effect of type of packaging on labeling^∗^transparency seems to be more robust with jar than with bag types of packaging. Note also that the behavior of the estimated marginal means of lightness and cookies liking ratings (being these ratings the only ones showing significant effects in the two types of packaging) tends to be somewhat similar in both, jar and bag.

#### Moderating Effect of Labeling on Type^∗^Transparency of Packaging

**FIGURE 6:**
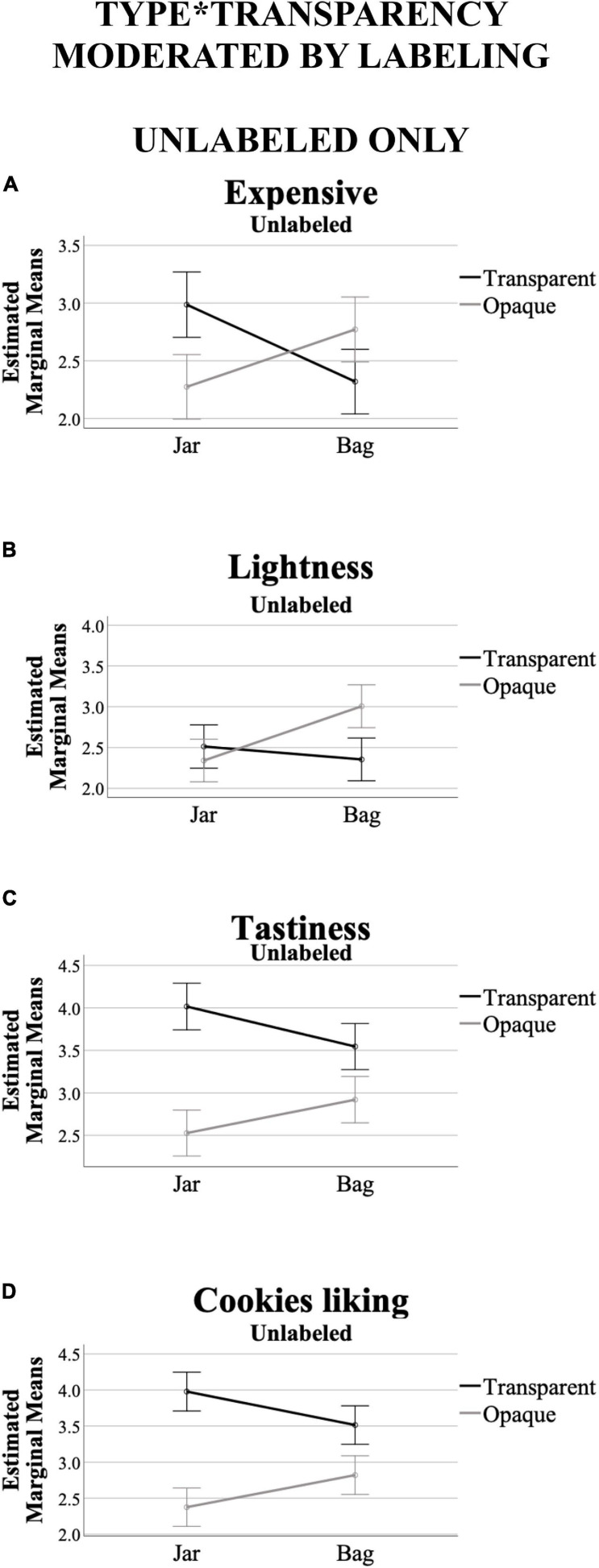
Interaction effect of type*transparency of packaging, while moderated by labeling (labeled vs. unlabeled), across the variables that prompted significant differences at 95% confidence in “Moderating Effect of Labeling on Type*Transparency of Packaging” [only for unlabeled, **(A)** expensive, **(B)** lightness, **(C)** tastiness, **(D)** cookies liking ratings]. *Y*-axis are the marginal means. *X*-axis are packaging types. The black (darker) line corresponds to the transparent ratings, whereas the gray (clearer) line the opaque ones. Error bars show confidence interval at 95%.

A significant effect was found for type^∗^transparency interaction only for unlabeled versions of packaging (unlabeled – *F*[5,238] = 4.98, *p* < 0.001, η^2^ = 0.095; labeled – *F*[5,238] = 0.70, *p* = 0.625, η^2^ = 0.014). Here, the between participants test showed significant effects on expensive (*F*[1,242] = 16.63, *p* < 0.001, η^2^ = 0.064), lightness (*F*[1,242] = 9.52, *p* = 0.002, η^2^ = 0.038), tastiness (*F*[1,242] = 9,74, *p* = 0.002, η^2^ = 0.039), and cookies liking (*F*[1,242] = 11.18, *p* = 0.001, η^2^ = 0.044), ratings – healthiness *F*[1,242] = 1.70, *p* = 0.193, η^2^ = 0.007). The type^∗^transparency interactions moderated by labeling are also visualized in Figure 6. In summary, the moderating effect of labeling on type^∗^transparency is mostly prompting significant effects in the unlabeled versions of packaging.

### Summary of Results

The obtained results suggest that changes in the visual appearance of packaging, in terms of presence (vs. absence) of label, packaging type (jar vs. bag), and transparency (vs. opaqueness), modulate the expectations of dietary cookies in a digital environment (H1). For instance, concerning the main effects of each visual cue (labeling, type of packaging, transparency), the presence vs. absence of label in the packaging, as well as the ability, or not, of visually inspecting the cookies, had a significant effect on most of the ratings (e.g., H1a, H1c). Moreover, when it comes to the interaction effects, for example, there were moderating effects of type of packing on transparency, and on labeling^∗^transparency (H1b).

Note that, when the participants were asked if they were consumers of dietary cookies, only 35.1% answered that they actually were (*n* = 174). Therefore, we decided to split the original dataset across those that reported consuming such types of cookies, and those who reported not doing so, and re-run two independent analyses. However, after running such analyses, no remarkable differences in the data were observed suggesting that such contrast in consumption habits triggered robust differences across the obtained results (cf. Lwin et al., 2014). Such null effects may also be related to the split of the sample and, hence, a consequent underpowered analysis.

## Discussion

The general purpose of this study was to evaluate the role of packaging format, label, and transparency on dietary products expectations. In particular, we aimed at looking for ways to customize the digital experience of dietary cookies more accurately via its packaging. For this, we assessed whether different visual appearances of packaging (labeled vs. unlabeled, bag vs. jar, transparent vs. opaque), would modulate the expectations for dietary cookies when presented in a partial simulation of e-commerce environment. Our results suggest that such different presentations of packing can indeed alter specific aspects of the expectations of consumers when evaluating dietary cookies (H1).

### The Role of Packaging Labeling When Looking for Dietary Cookies in Digital Environments (H1a)

As expected, the presence of labeling in the packaging primed the consumers toward a more precise understanding of the dietary characteristics of such cookies, when compared to unlabeled versions of the same packaging [De Luca et al., 2019; see section “Main Effect of Packaging Labeling (With vs. Without Label)”]. Hence, the short and precise information present in this experimental and customized label was able to effectively nudge the consumer toward the understanding of the principal dietary characteristics of the product at stake (i.e., Carrillo et al., 2012). What is more, even when the label did not specify any precisions regarding the sensory characteristics of the cookies, the presence of label also enhanced the ratings related to greediness, crumbliness, sweetness, tastiness, cookies, and packaging liking (cf. Pinto et al., 2017). Note that the cookies with labeling were also rated as the most expensive. Hence, we could further hypothesize that the presence of label gave support to a more meticulous framing of the cookies within a specific category (i.e., dietetic food), where prices are usually above the average when compared to the generic version of the same product.

### The Role of Type of Packaging When Looking for Dietary Cookies in Digital Environments (H1b)

There was no evidence of a main effect of type of packaging on expectations. However, while assessing the interactions, there were moderating effects of type of packing on transparency, and on labeling^∗^transparency. For instance, as shown in section “Interaction Effect Between Type and Transparency,” people tend to expect cookies to be more expensive when presented in a transparent jar, as compared to a transparent bag. Moreover, bag ratings seem to be generally less affected by changes in transparency, when compared to the jar. Hence, and even though type of packaging did not prompt as robust main effects, when compared to labeling and transparency, it can still be of importance to consider the type of packaging in order to get more precise effects out of these other two visual cues, and especially when it comes to combining type of packaging with transparency.

### The Role of Packaging Transparency When Looking for Dietary Cookies in Digital Environments (H1c)

The results show that a transparent vs. opaque packaging can generally trigger higher expectations concerning the overall quality (which is consistent with Sabri et al., 2020), but not necessarily when it comes to expected healthiness, [see section “Main Effect of Packaging Transparency (Transparency vs. Opaqueness)”]. In particular, we saw that the transparent packaging generally prompted higher expectations with concerns to aspects that could be understood as qualitative (think of a product’s quality, liking for the product and packaging, greediness), as well as sensory ones (crumbliness, sweetness). On the other hand, however, the transparency of the packaging had an opposite effect when it comes to the perceived healthiness, where the cookies were expected to be less light, and higher in calories, when presented in a transparent (vs. opaque) packaging. In fact, when considering the interaction effects reported in “Interaction Between Labeling and Transparency,” the unlabeled and transparent packaging was expected to provide sweetest cookies when compared to the unlabeled, but opaque, presentation. Hence, we could conclude that allowing the visual inspection of the cookies can encourage the consumer toward more hedonic – thus, potentially less rational – choices during decision-making tasks related to the online consumption of dietary food (Simmonds et al., 2018), and that this might be independent of the information that the brand can deliver to the consumer via labeling (cf. Adam and Ali, 2014). The latter may also be in line with the fact that transparent packaging can lead to better imagery, thus eating simulation could be the mechanism explaining such an effect (Petit et al., 2021).

### General Implications

A thoroughly customized packaging experience can have important consequences on the way a consumer interacts with a product. Based on the particular results of this study, it may be possible to more effectively nudge consumers toward healthier choices of cookies, while at the same time allowing brands of dietary cookies to better market such type of products. For instance, as shown in this study, if a brand of cookies is looking for to emphasize its dietary characteristics, they might as well prioritize communicating such attributes via effective labeling [as in section “Main Effect of Packaging Labeling (With vs. Without Label)”], and perhaps not rely on transparent packaging. However, another brand that may be more interested in evoking, say, greediness during decision-making tasks, could perhaps focus on the design of the cookies, and show them via transparent packaging [as in section “Main Effect of Packaging Transparency (Transparency vs. Opaqueness)”].

The obtained results are certainly relevant in a digital environment where vision (and to a lesser extent audition and touch), tends to be the principal sense (Petit et al., 2019). As a matter of fact, a profound customization of the visual aspects of packaging may be a good start when considering the future impact of new technologies on the stages of the consumer’s journey where, for instance, further advances in virtual/augmented reality will certainly transform the online shopping experience (Hoyer et al., 2020).

From a marketing perspective, this study also highlights how a food company – in an online environment – can work on its own identity by strengthen packaging communication. For instance, the obtained evidence offers packaging design solutions for safeguarding brand awareness, along with the possibility of enhancing quality and healthiness expectations during e-commerce decision-making tasks.

### Limitations and Future Directions

Concerning the study’s limitations, only visual aspects of packaging were tested. Therefore, future similar studies could compare, not only packaging aspects, but also different appearance of cookies. When it comes to the particular visual aspects of packaging that were tested, future studies could further manipulate packaging material, where sensations related to smoothness vs. roughness could be further enhanced (e.g., while retesting H1b). In this study we only tested presence vs. absence of customized labeling. However, the customization of labeling in dietary cookies could also be further addressed in similar future studies by, e.g., testing different combinations of messages along with particular color and/or font customization. Here we only tested one type of transparency in packaging as well. It may be of use to compare in the future glossy vs. matte transparent cookie jars, as it has been suggested that such differences can affect the perception of a packaging in terms of, e.g., haptics (Decré and Cloonan, 2019). Important to note as well that this study has the particular limitation of not considering that the choice of a packaging’s type is not only about aesthetics, but it might also be related to the performance of such packaging across the corresponding supply chain.

In brief, and even though this assessment has the abovementioned limitations, this study invites toward continuing exploring the further customization of packaging for dietary product categories, and especially considering potential new consumers of these type of products in digital environments.

## Data Availability Statement

The original contributions presented in the study are included in the article/supplementary material, further inquiries can be directed to the corresponding author/s.

## Ethics Statement

Ethical review and approval was not required for the study on human participants in accordance with the local legislation and institutional requirements. The patients/participants provided their written informed consent to participate in this study.

## Author Contributions

FR-C, RC, and MD: conceptualization. FR-C, RC, MD, and CV: methodology. FR-C and CV: formal analysis. FR-C: original draft preparation and funding acquisition. FR-C, RC, and CV: writing—review and editing. All authors contributed to the article and approved the submitted version.

## Conflict of Interest

The authors declare that the research was conducted in the absence of any commercial or financial relationships that could be construed as a potential conflict of interest.

## Footnotes

1 Data Osservatorio Doxa/Union Food; https://unionfood.iocominciobene.inc-press.com/; retrieved 13th of May 2021.

2 In order to determine the sample size, a power analysis was performed based on Friedman’s simplified determinations of statistical power (see Friedman, 1982, Table 1). Considering 95% confidence (α = 0.05), effect size (*r*) of 0.35, and a power effect of at least 0.80, the suggested sample size would more than 59 participants per between participants’ condition.

3 qualtrics.com

4 https://app.prolific.co/

## Appendix

**Appendix Table A:** Summary of experimental design, including the variables sampled, and the system used to measure each variable.

Section of Questionnaire	Variable	Measurement system
Ratings Scales	I expect these cookies to be healthy	5-points rating scales (1 ‘Not at all’; 5 ‘Very much’)
	(Healthiness)	
	I expect these cookies to be of good quality	
	(Quality)	
	These cookies look so good they make me hungry	
	(Greediness)	
	I expect these cookies to be expensive	
	(Expensive)	
	I expect these cookies to be light	
	(Lightness)	
	I expect these cookies to be low in calories	
	(Low in calories)	
	I expect these cookies to be high in calories	
	(High in calories)	
	I expect these cookies to be crumbly	
	(Crumbliness)	
	I expect these cookies to be sweet	
	(Sweetness)	
	I expect these cookies to be tasty	
	(Tastiness)	
	I expect these cookies to be good	
	(Cookies liking)	
	I like this packaging	
	(Package liking)	
Demographics, basic cookies habits	Age	Open question
	Gender	Male; female; other
	I eat cookies	Yes; No
	I love cookies	
	I consume light and/or dietary cookies	
	I usually pay attention to the information on a food/drink product’s label	

*The presentation of the variables was fully randomized.*
